# Association of Cardiovascular Burden with Mobility Limitation among Elderly People: A Population-Based Study

**DOI:** 10.1371/journal.pone.0065815

**Published:** 2013-05-31

**Authors:** Anna-Karin Welmer, Sara Angleman, Elisabeth Rydwik, Laura Fratiglioni, Chengxuan Qiu

**Affiliations:** 1 Aging Research Center (ARC), Department of Neurobiology, Care Sciences and Society, Karolinska Institutet and Stockholm University, Stockholm, Sweden; 2 Karolinska University Hospital, Stockholm, Sweden; 3 Research and Development Unit, Jakobsbergs Hospital, Järfälla, Sweden; 4 Stockholm Gerontology Research Center, Stockholm, Sweden; The University of Manchester, United Kingdom

## Abstract

**Background:**

Cardiovascular risk factors (CRFs) such as smoking and diabetes have been associated with mobility limitations among older adults. We seek to examine to what extent individual and aggregated CRFs and cardiovascular diseases (CVDs) are associated with mobility limitation.

**Methods:**

The study sample included 2725 participants (age ≥60 years, mean age 72.7 years, 62% women) in the Swedish National Study on Aging and Care in the Kungsholmen district of central Stockholm, Sweden, who were living either at their own home or in institutions. Data on demographic features, CRFs, and CVDs were collected through interview, clinical examination, self-reported history, laboratory tests, and inpatient register. Mobility limitation was defined as walking speed <0.8 m/s. Data were analyzed using multiple logistic models controlling for potential confounders.

**Results:**

Of the 2725 participants, 581 (21.3%) had mobility limitation. The likelihood of mobility limitation increased linearly with the increasing number of CRFs (i.e., hypertension, high C-reactive protein, obesity, diabetes and smoking) (*p* for linear trend<0.010) and of CVDs (i.e., ischemic heart disease, atrial fibrillation, heart failure and stroke) (*p* for linear trend<0.001). There were statistical interactions of aggregated CRFs with age and APOE ε4 allele on mobility limitation (*p*_interaction_<0.05), such that the association of mobility limitation with aggregated CRFs was statistically evident only among people aged <80 years and among carriers of the APOE ε4 allele.

**Conclusion:**

Aggregations of multiple CRFs and CVDs are associated with an increased likelihood of mobility limitation among older adults; however the associations of CRFs with mobility limitation vary by age and genetic susceptibility.

## Introduction

Walking speed is a reliable marker of mobility and an independent predictor of disability and cognitive decline [1], [2]. Cardiovascular risk factors (CRFs), such as smoking, hypertension, hypercholesterolemia, diabetes mellitus, and obesity, are considered major risk factors for cardiovascular diseases (CVDs) including cerebrovascular disease [3], [4]. In addition, epidemiological studies have suggested that high C-reactive protein as a marker of inflammation is associated with an increased risk for CVDs [5]. Since, slow walking speed may be a clinical manifestation of cardiovascular and cerebrovascular diseases [6], identifying the potentially modifiable factors may provide an opportunity for prevention at an early stage of mobility limitations. Indeed, CRFs are known to cause cerebrovascular lesions, which may contribute to common geriatric syndromes such as impaired cognitive and physical functions [7]. For example, smoking has been associated with slower walking speed in older people [8]. Hypertension has been associated with slower walking speed and higher decline in walking speed in the French Three-City Study [9]. Although high total cholesterol is associated with adverse health outcomes [10], [11], its association with walking speed among older people remains uncertain. A substantial amount of physical disability has been attributed to diabetes, even among physically impaired older women [12]. Both obesity and being underweight are independent predictors of slow walking speed and physical disability in middle-aged and older people [13], [14]. Increasing levels of inflammatory markers have been found to be predictive of mobility limitation [15]. Most previous studies examining the association of CRFs and CVDs with mobility limitation have focused on individual factors. However, these factors frequently coexist as people age. Studying the association of clustering CRFs and CVDs with impaired walking speed will help to better understand the impact of cardiovascular burden on mobility limitation in older people.

An age-dependent association of CRFs with physical limitation has been suggested for certain CRFs (e.g. hypertension and obesity), such that having these CRFs in younger-old age is more strongly associated with physical limitation compared to having these factors in late-life [13], [16]. In addition, gender differences in the association of CRFs and CVDs with adverse health outcomes have been reported [17], suggesting that these factors may affect mobility differently in men and women. Finally, previous studies have suggested that the APOE ε4 allele may modify the associations of certain CRFs, e.g. smoking and diabetes, with cognitive impairment [18], [19]. It remains, however, to be elucidated whether demographic factors or the APOE ε4 allele may modify the association of clustering CRFs and CVDs with mobility limitation.

On the basis of previous research, we hypothesize that CRFs or CVDs are associated with mobility limitation owing to their contribution to cerebrovascular lesions. In this population-based study of older men and women, we seek to test this hypothesis by examining (1) to what extent individual and aggregated CRFs and CVDs are associated with mobility limitation, and (2) whether the associations vary by age, gender or APOE ε4 allele.

## Materials and Methods

### Ethics Statement

The SNAC-K study was approved by the Ethics Committee at Karolinska Institutet and by the Regional Ethical Review Board in Stockholm (number: KI 01-114). Informed written consent was obtained directly from participants, or in case of cognitively impaired persons, from informants. Research within SNAC-K had been conducted according to the principles expressed in the Declaration of Helsinki.

### Subjects

Participants were taken from the Swedish National Study on Aging and Care in Kungsholmen (SNAC-K), which is one of four subprojects included in the national study [20]. The SNAC-K study population consists of a sample of people aged ≥60 years, living at their own home or in institutions in Kungsholmen, an area of central Stockholm, Sweden. The study used stratified sampling by age; the population was first stratified by age, and then a random sample was selected from each selected age group. In total, eleven age cohorts were chosen with two different age intervals: intervals of six years in the younger cohorts (60, 66, 72 and 78 years), and three years in the older cohorts (81, 84, 87, 90, 93, 96 and 99+ years). The data collection was conducted during March 2001–June 2004. A total of 5111 persons were selected for participation. Of these, 4590 were alive and eligible to participate, and 3363 (73.3%) participated at the baseline examination. We excluded 184 persons with missing information on walking speed, and 454 persons with missing information on blood pressure (n = 78), C-reactive protein (n = 265), body mass index (n = 258) and smoking (n = 86), leaving 2725 subjects for the current analyses. Compared to the excluded subjects (n = 638), the analytical sample was significantly younger (mean age ± SD, 72.7±10.2 vs. 83.6±11.2 years, p<0.001), included fewer women (61.8% vs. 78.2%, p<0.001), and had a higher level of education (for university education, 35.7% vs. 19.5%, p<0.001).

### Data Collection

Data was collected at our research center via interviews, clinical examinations, and psychological testing by trained staff. For those who agreed to participate but were unable or unwilling to come to the research center, home visits were conducted. We collected data on demographics (age, gender, and education), CRFs and CVDs (e.g., hypertension, obesity, smoking, diabetes and heart disease), use of medications (e.g., antihypertensive, blood glucose-lowering agents), and APOE genotype. Education was measured as the highest level of formal education and was categorized as elementary school (grade 1–9), high school (grade 10–12), or university or above. Medical conditions were diagnosed by the examining physicians based on the clinical examination, self-reported medical history and laboratory data. Information on medical history was also taken from the computerized inpatient register system that encompassed all hospitals in Stockholm since 1969. The International Classification of Diseases-10th Revision (ICD-10) was used to classify diseases. Medications were grouped according to the Anatomical Therapeutic Chemical (ATC) classification system. Genomic DNA was extracted from peripheral blood samples and a standard procedure was used for APOE genotyping [21]. Data on APOE genotype were available for 2494 (91.5%) subjects.

#### Definition of mobility limitation

*Walking speed* was assessed by trained nurses, where participants were requested to walk 2.4 or 6 meters a self-selected speed [22]. The length of the walk was determined by asking the participants how fast they normally walk. Subjects who rated themselves as fast or normal walkers did the longer walk and slow or very slow self-rated walkers did the shorter walk. At home visits, the shorter walk was always conducted due to space restrictions. For the analyses, the walking speed reflects the time from whichever walk was performed by the participant. Subjects who were unable to walk without personal support received the worst possible score, i.e. 0 m/s. Mobility limitation was defined as a walking speed <0.8 m/s because it may reflect underlying disease [1], [2].

#### Assessment of cardiovascular risk factors (CRFs)

*Smoking status* was categorized into never, former or current smoking. *Arterial blood pressure* (systolic Korotkoff phase I and diastolic phase V) was measured twice on the left arm with a 5-minute interval using a sphygmomanometer in the sitting position after at least a 5-minute rest. The mean of the two readings was used in defining hypertension status, which was categorized as normal blood pressure (<120/80 mm Hg), prehypertension (120–139/80–89 mm Hg, reference), hypertension stage 1 (140–159/90–99 mm Hg), and hypertension stage 2 (≥160/100 mm Hg or use of antihypertensive agents [ATC codes: C02, C03 and C07-C09]) [23].

We first measured non-fasting serum cholesterol. If total cholesterol level was ≥6.5 mmol/L, then fasting serum cholesterol was measured. *High cholesterol* was defined as a fasting total cholesterol level ≥6.5 mmol/L.

*Diabetes* was ascertained based on self-reported history of diabetes, having a diagnosis of diabetes in the inpatient register, current use of oral glucose-lowering agents or insulin injection (ATC code A10), or having a glycosylated hemoglobin level ≥5.4% [24].

*Body mass index (BMI)* was calculated by dividing weight in kilograms by height in meters squared, and was categorized as underweight (<20), normal (20–24.9, reference), overweight (25–29.9) and obesity (≥30 kg/m^2^) [25].

*C-reactive protein (CRP)* concentrations in plasma were measured following a standard procedure. High CRP was defined as CRP levels higher than 5 mg/L [5].

#### Ascertainment of cardiovascular diseases (CVDs)

*Ischemic heart disease, atrial fibrillation, heart failure and stroke* were defined based on clinical examination, electrocardiogram, self-reported medical history, or inpatient register information (ICD-10 codes I20-I25 for ischemic heart disease, I48 for atrial fibrillation, I50 for heart failure, and I60-I69 for stroke).

### Data Analysis

Descriptive analysis was performed to show the distribution of demographic and clinical characteristics of the study participants by mobility status. Statistical differences were examined using *chi*-square test or t-test. Logistic regression analyses were conducted to estimate the odds ratio (OR) and 95% confidence interval (CI) of mobility limitation associated with individual factors and clustering CRFs or CVDs. The aggregated CRFs included stage 2 hypertension, high CRP, obesity, diabetes and smoking, and the aggregated CVDs consisted of ischemic heart disease, atrial fibrillation, heart failure and stroke. The aggregations were defined by counting the number of CRFs and CVDs, separately, and were categorized into 0, 1, and ≥2 CRFs or CVDs. We did not include high total cholesterol in the aggregation of CRFs because high cholesterol showed a strong association with reduced odds of mobility limitation. We reported results from two main models: In model 1 individual CRFs or CVDs were analyzed separately, adjusting for age, gender and education. In model 2 all CRFs and CVDs were included in the same model, along with adjustment for demographics and APOE genotype. Statistical interactions were tested by including simultaneously the independent variables and their cross-product variables in the same model. We further performed stratified analysis when statistical interactions were detected.

The effect of missingness for the CRFs (n = 454) on mobility limitation was evaluated with multiple imputation technique (multiple imputation chained equations) [26]. All relevant variables included in the analyses were used in the multiple imputation models, including the outcome. The STATA 12 software was used for all analyses.

## Results

Of the 2725 participants, 581 (21.3%) were identified to have mobility limitation. Subjects with mobility limitation were older, more often women, less educated, more likely to have stage 2 hypertension, high CRP, diabetes, ischemic heart disease, atrial fibrillation heart failure and stroke, less likely to have high total cholesterol and to smoke and were more often underweight compared with subjects without mobility limitation (Table 1).

**Table 1:** Characteristics of the study participants by mobility status (n = 2725).

	Mobility limitation		
Characteristics	No (n = 2144)	Yes (n = 581)	*p*-value
Age (years), mean (SD)	70.1 (8.9)	82.5 (8.7)	<0.001
Female, n (%)	1260 (58.8)	423 (72.8)	<0.001
Educational level, n (%)			
Elementary school	241 (11.2)	161 (27.7)	
High school	1044 (48.7)	307 (52.8)	
University or above	859 (40.1)	113 (19.5)	<0.001
APOE ε4-carriers*, n (%)	590 (29.8)	138 (27.0)	0.223
Hypertension status, n (%)			
Nomotension	118 (5.5)	31 (5.3)	
Prehypertension	481 (22.4)	73 (12.6)	
Stage 1 hypertension	545 (25.4)	100 (17.2)	
Stage 2 hypertension	1000 (46.6)	377 (64.9)	<0.001
High cholesterol (≥6.5 mmol/L), n (%)	298 (13.9)	35 (6.0)	<0.001
High C-reactive protein (>5 mg/L), n (%)	351 (16.4)	171 (29.4)	<0.001
Body mass index (kg/m^2^), n (%)			
<20 (underweight)	76 (3.5)	88 (15.2)	
20–24.9 (normal weight)	893 (41.7)	228 (39.2)	
25–29.9 (overweight)	901 (42.0)	189 (32.5)	
≥30 (obesity)	274 (12.8)	76 (13.1)	<0.001
Smoking status, n (%)			
Never	933 (43.5)	318 (54.7)	
Former	881 (41.1)	199 (34.3)	
Current	330 (15.4)	64 (11.0)	<0.001
Diabetes, n (%)	171 (8.0)	83 (14.3)	<0.001
Ischemic heart disease, n (%)	301 (14.0)	185 (31.8)	<0.001
Atrial fibrillation, n (%)	252 (11.8)	181 (31.2)	<0.001
Heart failure, n (%)	119 (5.6)	177 (30.5)	<0.001
Stroke, n (%)	57 (2.7)	68 (11.7)	<0.001

* Data were missing for 231 subjects.

Table 2 shows the association of individual CRFs and CVDs with mobility limitation. In model 1, normal blood pressure, high CRP, underweight, obesity, diabetes, ischemic heart disease, atrial fibrillation, heart failure and stroke were significantly associated with an increased OR of mobility limitation, whereas high cholesterol was associated with reduced OR of mobility limitation. In model 2, the associations of obesity and ischemic heart disease with mobility limitation were attenuated and no longer significant (Table 2). The APOE ε4 allele was not significantly associated with mobility limitation.

**Table 2:** Odds ratios (95% confidence interval) of mobility limitation associated with cardiovascular risk factors and vascular diseases (n = 2725).

Vascular risk factors and cardiovascular diseases	Odds ratio (95% confidence interval)	
	Model 1*	Model 2*
Hypertension status		
Nomotension	2.00 (1.13–3.54)	1.98 (1.10–3.58)
Prehypertension	1.00 (Reference)	1.00 (Reference)
Stage 1 hypertension	0.90 (0.60–1.34)	1.00 (0.65–1.52)
Stage 2 hypertension	1.19 (0.85–1.67)	0.92 (0.63–1.33)
High cholesterol (≥6.5 mmol/L)	0.32 (0.21–0.50)	0.37 (0.23–0.58)
High C-reactive protein (>5 mg/L)	1.97 (1.49–2.61)	1.78 (1.32–2.40)
Body mass index (kg/m^2^)		
<20 (underweight)	3.15 (2.05–4.83)	2.79 (1.80–4.34)
20–24.9 (normal weight)	1.00 (Reference)	1.00 (Reference)
25–29.9 (overweight)	1.16 (0.89–1.53)	1.16 (0.87–1.55)
≥30 (obesity)	1.68 (1.12–2.51)	1.38 (0.90–2.11)
Smoking status, n (%)		
Never	1.00 (Reference)	1.00 (Reference)
Former	0.96 (0.74–1.25)	0.87 (0.66–1.15)
Current	1.17 (0.80–1.73)	1.11 (0.75–1.66)
Diabetes	1.44 (1.19–1.74)	1.31 (1.08–1.58)
Ischemic heart disease	1.60 (1.22–2.09)	1.20 (0.88–1.63)
Atrial fibrillation	2.27 (1.72–3.00)	1.56 (1.14–2.14)
Heart failure	3.16 (2.29–4.37)	2.11 (1.45–3.05)
Stroke	3.47 (2.17–5.55)	3.31 (2.05-5.36)
APOE ε4 allele	1.09 (0.84–1.43)	1.10 (0.83–1.46)

* Model 1 was adjusted for demographics, and Model 2 included all vascular factors and cardiovascular diseases as well as demographics and APOE genotype.

For the clustering of CRFs where stage 2 hypertension, high CRP, obesity, diabetes and smoking were aggregated, we found that the likelihood of mobility limitation increased linearly with increasing number of CRFs, when adjusting for demographics and APOE genotype (Table 3, model 1). The association of aggregated CRFs with mobility limitation was attenuated, but remained statistically significant when CVDs were further included in the model (Table 3, model 2).

**Table 3:** Odds ratios (95% confidence interval) of mobility limitation associated with clustering of CRFs and CVDs in the total sample (n = 2725).

	No. of	No. of	Odds ratio (95% confidence interval)	
Numbers of CRFs and CVDs	subjects	cases	Model 1*	Model 2*
CRFs†				
0	830	109	1.00 (Reference)	1.00 (Reference)
1	1093	234	1.27 (0.94–1.72)	1.09 (0.80–1.50)
≥2	802	238	1.97 (1.43–2.71)	1.57 (1.13–2.19)
*p* for linear trend			<0.001	0.010
CVDs‡				
0	1847	234	1.00 (Reference)	1.00 (Reference)
1	526	161	1.99 (1.49–2.67)	1.93 (1.43–2.59)
≥2	352	186	3.55 (2.59–4.85)	3.28 (2.39–4.50)
*p* for linear trend			<0.001	<0.001

CRF  =  cardiovascular risk factor; CVD  =  cardiovascular disease.

* Model 1 was adjusted for demographics and APOE genotype, and Model 2 included CRF and CVDs as well as demographics and APOE genotype.

† The aggregation of CRFs included stage 2 hypertension, high C-reactive protein, obesity, diabetes and smoking.

‡ The aggregation of CVDs included ischemic heart disease, atrial fibrillation, heart failure and stroke.

For the clustering of CVDs where ischemic heart disease, atrial fibrillation, heart failure and stroke were aggregated, having an increasing number of CVDs was strongly associated with increased OR of mobility limitation (*p* for linear trend <0.001 in both model 1 and 2).

We detected statistical interactions of aggregated CRFs with age (<80 vs. ≥80 years) and APOE ε4 allele (*p*_interaction_ ≤0.05) on mobility limitation. Further analyses stratifying by age or APOE ε4 allele were performed. The age-stratified analyses suggested that the significant association of aggregated CRFs with mobility limitation was only present among people aged <80 years; the association with mobility limitation was particularly strong for having multiple (≥2) CRFs, even when CVDs were controlled for (Table 4).

**Table 4:** Odds ratios (95% confidence interval) of mobility limitation associated with clustering of CRFs by age.

	Age <80 years (n = 2028)			Age ≥80 years (n = 697)		
	No. of	Odds ratio (95% confidence interval)		No. of	Odds ratio (95% confidence interval)	
Number of CRF*	subjects/cases	Model 1†	Model 2†	subjects/cases	Model 1†	Model 2†
0	672/35	1.00 (Reference)	1.00 (Reference)	158/74	1.00 (Reference)	1.00 (Reference)
1	789/64	1.14 (0.73–1.79)	1.00 (0.63–1.58)	304/170	1.33 (0.88–2.02)	1.15 (0.74–1.78)
≥2	567/107	3.40 (2.23–5.18)	2.68 (1.74–4.12)	235/131	1.39 (0.89–2.16)	1.14 (0.72–1.81)
*p* for linear trend		<0.001	<0.001		0.293	0.806

CRF  =  cardiovascular risk factor.

* The aggregation of CRFs included stage 2 hypertension, high C-reactive protein, obesity, diabetes and smoking.

† Model 1 was adjusted for demographics and APOE genotype, and in Model 2 additional adjustment was made for CVDs.

Analyses stratified by APOE ε4 status revealed that the significant association of aggregated CRFs with mobility limitation was present only among carriers of the APOE ε4 allele (Table 5).

**Table 5:** Odds ratios (95% confidence interval) of mobility limitation associated with clustering of CRFs by APOE ε4 status.

	Non-carriers of APOE ε4 allele (n = 1766)			Carriers of APOE ε4 allele (n = 728)		
	No. of	Odds ratio (95% confidence interval)		No. of	Odds ratio (95% confidence interval)	
Number of CRF*	subjects/cases	Model 1†	Model 2†	subjects/cases	Model 1†	Model 2†
0	543/73	1.00 (Reference)	1.00 (Reference)	225/21	1.00 (Reference)	1.00 (Reference)
1	689/148	1.08 (0.74–1.57)	0.92 (0.62–1.36)	312/62	2.32 (1.17–4.61)	1.88 (0.93–3.81)
≥2	534/152	1.67 (1.13–2.47)	1.30 (0.86–1.96)	191/55	3.98 (1.87–8.44)	3.17 (1.48–6.76)
*p* for linear trend		0.012	0.136		0.002	0.011

CRF  =  cardiovascular risk factor.

* The aggregation of CRFs included stage 2 hypertension, high C-reactive protein, obesity, diabetes and smoking.

† Model 1 was adjusted for demographics and APOE genotype, and in Model 2 additional adjustment was made for CVDs.

There was no statistical interaction of aggregated CVDs with age, gender or APOE ε4 allele status on mobility limitation (*p*_interaction_>0.10).

Finally, in the sensitivity analysis we excluded (1) people with CVDs or dementia (n = 893), and (2) people with underweight (n = 164) from the analytical sample, which yielded results similar to those presented in Tables 2–5 (data not shown). The multiple imputation analyses showed that the missing data on CRFs did not introduce any bias in our analyses. When subjects with imputed values were included in the analytical sample, similar results were obtained.

## Discussion

In this population-based study of people aged ≥60 years, we examined the association of cardiovascular burden with mobility limitation: when aggregating stage 2 hypertension, inflammation, diabetes, obesity and smoking, an increasing number of CRFs linearly increased the likelihood of mobility limitation; similarly, when aggregating ischemic heart disease, atrial fibrillation, heart failure and stroke, an increasing number of CVDs was strongly associated with an increased OR of mobility limitation. Furthermore, these associations with clustering of CRFs appear to vary by age and APOE ε4 status. Specifically, the association of aggregated CRFs with mobility limitation is only evident among people <80 years or among carriers of the APOE ε4 allele. Finally, our study also suggested that high cholesterol was associated with the reduced OR of mobility limitation in old age. These results support the involvement of vascular pathways in late-life mobility limitation.

For individual CRFs, we found that high levels of CRP were associated with mobility limitation, possibly by the role of inflammation in the aging-related process that leads to sarcopenia and loss of muscle strength [27]. Obesity was significantly associated with mobility limitation when adjusting for demographics. This association may be an adaptive response to the higher energy expenditure of walking for obese people. It may also partly be explained by the association of obesity with inflammation and sarcopenia [27]. Diabetes was significantly associated with mobility limitation, which might be explained by multiple pathways associated with diabetes such as peripheral vascular disease, peripheral neuropathy, impaired muscle strength and balance, visual limitation and inflammation [12], [28]. Blood pressure below 120/80 was associated with an increased OR of mobility limitation. This finding is consistent with previous studies that show an association of low and normal blood pressure with an increased risk for poor health outcomes among frail elderly people [29]. High total cholesterol was inversely associated with mobility limitation, which is in line with the theories of a bidirectional relation between total cholesterol and adverse health outcomes [10], [30], suggesting that low total cholesterol in late life may reflect frailty.

CRFs or CVDs often occur concurrently among older adults. However, few studies have investigated the association of clustered CRFs and cardiovascular multimorbidity with mobility limitation. A major finding in this study is that clustering of CRFs or CVDs was associated with mobility limitation. For the aggregated CRFs, we found that the likelihood of mobility limitation was increased linearly with increasing number of CRFs. When including CVDs in the model, the associations attenuated, suggesting that the association of aggregated CRFs with mobility limitation was partly mediated by CVDs. In line with previous research that demonstrates an age-dependent effect of hypertension and obesity on physical limitation [13], [16], our results suggest that the association of mobility limitation with the aggregated CRFs is only evident among people aged <80 years. The lack of association between aggregated CRFs and mobility limitation among people aged 80+ years may be explained by selected survival amongst the very old with mobility limitation [2], or by the possibility that non-CRFs play a larger role for mobility limitation among the oldest-old compared to younger-old people. Furthermore, our results extend previous research that reveals stronger associations of CRFs (e.g., smoking and diabetes) with cognitive decline amongst APOE ε4 carriers than non-carriers of the ε4 allele [18], [19], by showing that the association of aggregated CRFs with mobility limitation exists only amongst carriers of the APOE ε4 allele. A possible explanation is that APOE ε4-carriers may be more vulnerable to CRFs due to less effective neural protection and repair [31].

Of the major CVDs, stroke is known to cause physical disability. The association of mobility limitation with ischemic heart disease, atrial fibrillation and heart failure may be explained by decreased physiologic reserve associated with frailty [6], or by low oxygenation and atherosclerotic changes associated with cardiovascular disease and microvascular brain lesions [7]. More importantly, we found that an increasing number of CVDs was strongly associated with an increased odds ratio of mobility limitation; people with two or more CVDs had more than three-fold increased likelihood of mobility limitation, suggesting a strong link of cardiovascular multimorbidity to mobility limitation.

A major strength of this study refers to the large sample of community-based older people both living at their own home and in institutions. Moreover, CRFs and CVDs were assessed based on information from multiple sources, such as face-to-face interview, clinical examination, laboratory tests, and inpatient register system. We examined the cardiovascular burden for mobility limitation by aggregating CRFs and CVDs separately, on which very few studies have focused. Finally, we employed an objective approach to testing walking speed instead of self-reported measure. However, this study also has limitations. First, the results may have been biased by the different distances covered with the walking speed test. It was however not feasible to perform the longer walk at home visits due to the often restricted space. Excluding this group would probably have led to more biased results since the subjects assessed on home visits often are more disabled in comparison with the entire sample. In addition, data from the US National Health and Nutrition Survey (NHANES) showed that walking speed measured over the distances 2.4 and 6 meters are comparable [32]. Moreover, tests for walking speed are generally considered highly reliable, regardless of the distance [22], [33], [34]. Secondly, the cross-sectional design does not allow us to establish the temporality of the observed associations. For example, it is possible that mobility limitation may have contributed to underweight or obesity by causing physical inactivity. Furthermore, the cross-sectional design is subject to survival bias, meaning that some people with CRFs or CVDs may not have survived to the age to be included in the study. This may have led to underestimations of the true association of CRFs and CVDs with mobility limitation. Finally, the selection of major CRFs in this study was based on the research hypothesis, the cross-sectional nature of the study and on previous reports. We did not include physical inactivity because of its potentially inverse causality with mobility limitation in the cross-sectional design, i.e. people with mobility limitation may be more likely to be physically inactive. We did not include high total cholesterol in the aggregation of CRFs owing to its strong association with a decreased likelihood of mobility limitation, which is in accordance with previous reports [10], [30]. Longitudinal studies may help to further clarify the associations of these factors with late-life mobility limitation.

In conclusion, our study suggests that aggregations of multiple CRFs and CVDs are associated with mobility limitation among older people; the associations vary by age and genetic susceptibility. Specifically, the association of the aggregated CRFs with mobility limitation was only evident among people <80 years, and older people with CRFs may be more vulnerable to mobility limitation if they also carry the APOE ε4 allele. This study may help improve our understanding of the vascular involvement in mobility limitation, which is critical in the development of intervention strategies.
